# Phosphorylated OmpR Is Required for Type 3 Fimbriae Expression in *Klebsiella pneumoniae* Under Hypertonic Conditions

**DOI:** 10.3389/fmicb.2018.02405

**Published:** 2018-10-12

**Authors:** Tien-Huang Lin, Yeh Chen, Jong-Tar Kuo, Yi-Chyi Lai, Chien-Chen Wu, Chun-Fa Huang, Ching-Ting Lin

**Affiliations:** ^1^Department of Urology, Taichung Tzu Chi Hospital, Buddhist Tzu Chi Medical Foundation, Taichung, Taiwan; ^2^School of Post-Baccalaureate Chinese Medicine, Tzu Chi University, Hualian, Taiwan; ^3^Department of Biotechnology, Hungkuang University, Taichung, Taiwan; ^4^Department of Biological Science and Technology, China University of Science and Technology, Taipei, Taiwan; ^5^Department of Microbiology and Immunology, Chung-Shan Medical University, Taichung, Taiwan; ^6^Institute of Biochemistry and Molecular Biology, National Yang-Ming University, Taipei, Taiwan; ^7^School of Chinese Medicine, China Medical University, Taichung, Taiwan

**Keywords:** *Klebsiella pneumoniae*, OmpR, type 3 fimbriae, MrkHIJ, c-di-GMP signaling, biofilm

## Abstract

OmpR/EnvZ is a two-component system that senses osmotic signals and controls downstream gene expression in many species of *Enterobacteriaceae*. However, the role of OmpR/EnvZ in *Klebsiella pneumoniae* remains unknown. In this study, we found that production of MrkA, the major subunit of type 3 fimbriae, was decreased under hypertonic conditions. A deletion mutant of *ompR* and a site-directed mutant with a single amino acid substitution of aspartate 55 to alanine (D55A), which mimics the unphosphorylated form of OmpR, markedly reduced MrkA production under hypertonic conditions. These results indicate that *K. pneumoniae* type 3 fimbriae expression is activated by the phosphorylated form of OmpR (OmpR∼P). Although no typical OmpR∼P binding site was found in the P*_mrkA_* sequence, *mrkA* mRNA levels and P*_mrkA_* activity were decreased in the Δ*ompR* and *ompR*_D55A_ strains compared with the wild type (WT) strain, indicating that OmpR∼P mediates type 3 fimbriae expression at the transcriptional level. Previous reports have demonstrated that a cyclic-di-GMP (c-di-GMP) related gene cluster, *mrkHIJ*, regulates the expression of type 3 fimbriae. We found that both the *ompR* and *ompR*_D55A_ mutants exhibited decreased *mrkHIJ* mRNA levels, intracellular c-di-GMP concentration, and bacterial biofilm amount, but increased total intracellular phosphodiesterase activity in response to hypertonic conditions. These results indicate that OmpR∼P regulates type 3 fimbriae expression to influence *K. pneumoniae* biofilm formation via MrkHIJ and modulation of intracellular c-di-GMP levels. Taken together, we herein provide evidence that OmpR∼P acts as a critical factor in the regulation of the c-di-GMP signaling pathway, type 3 fimbriae expression, and biofilm amount in *K. pneumoniae* in response to osmotic stresses.

## Introduction

*Klebsiella* *pneumoniae* is a Gram-negative pathogen, which causes purulent abscess, bacteremia, and urinary as well as respiratory tract infections mostly in patients with underlying diseases (Podschun and Ullmann, 1998). Similar to many enteric bacteria, *K. pneumoniae* needs to sense various environmental signals, including temperature, nutrition-limitation, pH, oxygen availability, osmolality, as well as other stimuli, in order to achieve successful infection (Carpenter and Payne, 2014; Lustri et al., 2017). Bacterial two-component systems (TCSs), typically consisting of a sensor histidine kinase and a response regulator, play a crucial role in sensing environmental signals and subsequent regulation of gene expression at the transcriptional level (Stock et al., 2000; Laub and Goulian, 2007). In Gram-negative bacteria, the OmpR/EnvZ TCS can sense an osmotic signal to further control downstream gene expression including genes encoding outer membrane proteins and various virulence factors (Mizuno and Mizushima, 1990; Oshima et al., 2002). EnvZ, the sensor kinase, auto-phosphorylates a conserved histidine residue in response to extracellular osmolality and then transfers the phosphate group to aspartate residue 55 on the receiver domain of OmpR to exert DNA binding activity (Taylor et al., 1981; Igo and Silhavy, 1988; Delgado et al., 1993). In uropathogenic *Escherichia coli*, OmpR promotes bacterial survival in the murine urinary tract and growth in human urine (Schwan, 2009). In addition, deletion of *ompR* can increase type 1 fimbriae expression via activation of *fimB* transcription and phase-ON positioning of the *fimS* element under a high osmolality environment (Rentschler et al., 2013). In *E. coli*, OmpR increases biofilm amount by negatively regulating the expression of a flagellar regulator (Samanta et al., 2013). Likewise, *Yersinia enterocolitica* OmpR promotes biofilm formation, but positively regulates flagellar synthesis (Raczkowska et al., 2011). Furthermore, the important role of OmpR in the control of bacterial virulence has been demonstrated in many bacteria including *Salmonella* Typhimurium, *Shigella flexneri*, *Y. pestis*, and *Acinetobacter baumannii* (Dorman et al., 1989; Bernardini et al., 1990; Reboul et al., 2014; Tipton and Rather, 2016). However, the role of OmpR in *K. pneumoniae* pathogenesis remains unknown.

Multiple virulence factors have been shown to be involved in *K. pneumoniae* infection including capsular polysaccharide (), lipopolysaccharides (LPS), type 1 and type 3 fimbriae, iron-acquisition systems, porins, and antibiotic resistance determinants, e.g., carbapenemases, extended-spectrum β-lactamases, efflux systems, DNA gyrase, and topoisomerase IV (Paczosa and Mecsas, 2016; Lee et al., 2017). However, most of these virulence factors are processed or embedded in the cell envelope and enable bacteria to take up nutrients and adhere to diverse surfaces or niches within the human host (Wu and Fives-Taylor, 2001; Kawai et al., 2011). In addition, biofilm formation is considered a key factor in the development of nosocomial infections and increases bacterial resistance to antibiotics, thus hindering medical treatment (Murphy and Clegg, 2012). CPS, LPS, and fimbriae are involved in biofilm formation in *K. pneumoniae* (Schembri et al., 2005; Balestrino et al., 2008). Interestingly, the thick capsule of *K. pneumoniae* inhibits fimbriae activity and assembly (Schembri et al., 2005) and cross-regulated expression between fimbriae and CPS for efficient infection has been suggested. Of these factors, type 3 fimbriae are considered the major determinant of biofilm formation in *K. pneumoniae* (Di Martino et al., 2003; Jagnow and Clegg, 2003; Wu et al., 2012). Although most *K. pneumoniae* strains possess type 1 and 3 fimbriae, type 3 fimbriae are mainly expressed in heavily encapsulated *K. pneumoniae* strains, while the production of type 1 fimbriae is poor and phase-variable (Schembri et al., 2005; Struve et al., 2008). Therefore, elucidating the regulation of type 3 fimbriae is required to gain a deeper understanding of *K. pneumoniae* pathogenesis.

In *K. pneumoniae*, type 3 fimbriae are encoded by the *mrkABCDF* operon (Di Martino et al., 2003; Jagnow and Clegg, 2003; Wu et al., 2012). A cyclic di-GMP (c-di-GMP) related gene cluster, *mrkHIJ*, located downstream of the type 3 fimbrial genes, has been demonstrated to play a central role in regulating type 3 fimbriae expression (Wilksch et al., 2011; Murphy and Clegg, 2012; Wu et al., 2012; Yang et al., 2013). Reverse-transcription PCR analysis revealed that while *mrkHIJ* is transcribed as a polycistronic mRNA, *mrkJ* is also independently transcribed (Wu et al., 2012). MrkH possesses that a PilZ-domain that binds c-di-GMP to activate its own promoter activity and type 3 fimbriae expression (Wilksch et al., 2011; Yang et al., 2013; Tan et al., 2015). MrkI is a predicated LuxR-type transcriptional regulator to activate its own operon and type 3 fimbriae expression (Wu et al., 2012). MrkJ contains an EAL domain with functional c-di-GMP phosphodiesterase (PDE) activity for hydrolysis of c-di-GMP and further repression of type 3 fimbriae expression (Johnson and Clegg, 2010). Although MrkHIJ play a critical role in regulating type 3 fimbriae expression, the regulation of *mrkHIJ* remains unclear. c-di-GMP is a bacterial secondary messenger that modulates biofilm formation and the expression of various virulence factors (Tamayo et al., 2007; Jenal et al., 2017). The intracellular concentration of c-di-GMP in bacteria is modulated by di-guanylate cyclases (DGCs) and PDEs (Simm et al., 2004; Hengge, 2009). *K. pneumoniae* YfiN harbors DGC domain and has a positive role in the control of type 3 fimbriae expression (Wilksch et al., 2011), while YjcC possess a PDE domain and plays a negative role in type 3 fimbriae expression (Huang et al., 2013). In addition to c-di-GMP-related proteins, several transcriptional regulators have been reported to be involved in the control of type 3 fimbriae expression in *K. pneumoniae* including histone-like nucleoid-structuring protein (H-NS), CRP, and ferric uptake regulator (Fur) (Wu et al., 2012; Ares et al., 2016; Lin et al., 2016). Thus, the regulation of type 3 fimbriae in *K. pneumoniae* in response to different environmental stimuli is complicated.

In this study, we found that type 3 fimbriae expression in *K. pneumoniae* was decreased under hypertonic conditions. In addition, phosphorylated OmpR (OmpR∼P) activated type 3 fimbriae expression through the c-di-GMP signaling pathway and further affected biofilm amount in response to osmolality stresses.

## Materials and Methods

All chemicals and reagents were purchased from Sigma (St. Louis, MO, United States) unless otherwise stated. All enzymes were purchased from New England Biolabs unless otherwise stated. General molecular techniques, e.g., PCR and eletroporation, were performed according to standard protocols (Sambrook and Russell, 2000).

### Bacterial Strains, Plasmids, and Culture Conditions

All bacterial strains and plasmids used in this study are listed in **Table 1**. Primers used in this study are listed in **Table 2**. Each strain was grown overnight in Luria-Bertani (LB) medium (1% tryptone, 0.5% yeast extract, and 1% [∼170 mM] sodium chloride [NaCl]) (BD Difco^TM^) with respective antibiotics at 37°C for 16 h, and then 1/200 of overnight culture were inoculated into LB broth at concentrations of 0, 50, 200, or 400 mM of NaCl until the bacteria were grown after that to reach exponential phase (OD_600nm_ = 0.6–0.8). However, instead of 1% NaCl, we used LB medium supplemented with 2.32% NaCl (= 400 mM) to analyze the hypertonic effects in *K. pneumoniae*. The antibiotics used include ampicillin (100 μg/ml), kanamycin (25 μg/ml), streptomycin (500 μg/ml), and tetracycline (12.5 μg/ml).

**Table 1:** Bacterial strains and plasmids used in this study.

Strains or plasmids	Descriptions	Reference or source
***K. pneumoniae***		
CG43S3	CG43 Sm^r^	Lai et al., 2001
Δ*ompR*	CG43S3Δ*ompR*	This study
*ompR_D55A_*	CG43S3*ompR_D55A_*	This study
Δ*lacZ*	CG43S3Δ*lacZ*	Lin et al., 2006
Δ*lacZ-*Δ*ompR*	CG43S3Δ*lacZ*Δ*ompR*	This study
Δ*lacZ- ompR_D55A_*	CG43S3Δ*lacZompR_D55A_*	This study
***E. coli***		
BL21(DE3)	*F^-^ ompT hsdS_B_[r_B_^-^m_B_^-^]gal dcm* [DE3]	New England Biolabs
S17-1 *λ pir*	*hsdR recA pro* RP4-2 [Tc::Mu; Km::Tn*7*] [*λpir*]	Miller and Mekalanos, 1988
**Plasmids**		
yT&A	Ap^R^, TA PCR cloning vector	Yeastern
pKAS46	Ap^r^, Km^r^, Positive selection suicide vector, *rpsL*	Skorupski and Taylor, 1996
pACYC184	Cm^r^, Tc^r^, plasmid with *p15A* origin of replication	Chang and Cohen, 1978
pmrkAZ15	Cm^r^, 402 bp fragment containing the region upstream of *mrkA* cloned into placZ15	Lin et al., 2016
pompR	Cm^r^, 1079 bp fragment containing an *ompR* allele cloned into pACYC184	This study
pET30b-OmpR	Km^r^, 716 bp fragment encoding full-length *ompR* allele cloned into pET30b	This study

**Table 2:** Primers used in this study.

Primer	Sequence (5′ → 3′)	Enzyme cleaved	
GT254	GGGATCCAGACCGTCTGGGGTCT	*Bam*HI	
GT255	CTCTAGACAGCGGGTGCATACG	*Xba*I	
GT256	CGGATCCATCTGTTCGGCGTTC	*Bam*HI	
GT257	CGAGCTCTACCTGTTCGGCTCTGGC	*Sac*I	
GT284	GGGATCCGCTCTCCATCAATGCTAA	*Bam*HI	
GT285	GAGATCTGAAAGACGTAGCGTACAGC	*Bgl*II	
GT288	CGGATCCAGACAAAATGGAGGGAACCCTA	*Bam*HI	
GT290	GCAATAGCAACATTCTGATTGG		
GT336	CCATATGCAAGAGAATTATAAGATTCT	*Nde*I	
GT337	GAAGCTTGCCTTAGAACCGTCCGGGAC	*Hind*III	
GT356	ATGCTGCCGGGCGAAGATGGT		
GT357	TTCTAGATACGAATAAACAGCCA	*Xba*I	
GT363	CAGAGCCAGCACCATCAGGTGGA		
GT367	GACATCAATCTGAAAGGCCAG		
GT368	AGGGATCACCACTGCCAGAA		
CC323	GGATCCTGCATGCTGTTGCGGTCAC	*Bam*HI	
CC324	GGATCCGCGGTTGCCATTGCTGCAGAG	*Bam*HI	
SY01	GGGATCCGGTAGTCCATCTCCTGCTTCA	*Bam*HI	
SY02	GAAGCTTGACGAACAGCAAGGTGACG	*Hind*III	

**For qRT-PCR**	**Sequence (5′ → 3′)**	**TaqMan probes**	**Target**

RT11	GGTAGGGGAGCGTTCTGTAA	67	23S rRNA
RT12	TCAGCATTCGCACTTCTGAT		
RT29	TAAGCAAACTGGGCGTGAA	20	*mrkA*
RT30	TAGCCCTGTTGTTTGCTGGT		
GT46	GTTTAAGTTCCGCCATCTCG	120	*mrkH*
GT47	TTGCGCTTGGCTTCTAAGAT		
GT42	AGTTATGCCGATGTCATCCAT	59	*mrkI*
GT43	GATTCTGATGGCAGAAATATCCTT		
GT54	TTTCGAGGTAACCGAAAACG	84	*mrkJ*
GT55	GAGGTATCCTGTGGGCTCTG		
RT159	CTGGCTGGATGATTTTGGTC	67	D364_06025
RT160	CCACTTTGACGCAATCGAA		
RT161	CGTAGGCTGGCTGATGGA	40	D364_08130
RT162	TCTGTCTCGGTGACCTCGAT		
RT164	GGGTTTGCTGATATCGATGG	67	D364_13295
RT165	AGCCAACGTTTTGACATGCT		
RT169	ATCGATTTTCGCCATCAGAG	70	*fimK*
RT170	GAAAAGCAGTCGTCCAGCAT		
RT175	AATGGTCATCCGGGAAGC	20	*yjcC*
RT176	CCGCTGAACACAACTCACC		
RT244	GCATCGTAACCTGGTCTTCAA	36	D364_02175
RT245	GAGCAGGCTGGTGAAAATG		
RT273	CCAGGATCCTCGCCACTAT	22	D364_04060
RT274	TCCCGACCTTACCAAAACG		
RT275	CGCCGGTACTGATCCAAT	28	D364_11845
RT276	ATTCACCACCAGCATCACG		
RT277	GCTACCTGGTGCGCAATATAA	68	D364_16830
RT278	TCGCCATAAATACAGCGTAGC		

### Construction of the *ompR* Deletion Mutant and Its Complementation

A specific *ompR* gene deletion was introduced into *K. pneumoniae* CG43S3 using an allelic exchange strategy as previously described (Lai et al., 2003). Briefly, the upstream and downstream regions (approximately 1000 bp DNA fragments) of *ompR* were cloned into suicide vector pKAS46 containing *rpsL*, which allows positive selection for the loss of vector with streptomycin (Skorupski and Taylor, 1996). The resulting plasmid was then mobilized from *E. coli* S17-1λ*pir* (Miller and Mekalanos, 1988) to *K. pneumoniae* CG43S3 or CG43S3-derived strains by conjugation. Overnight cultures of donor and recipient strains were mixed in a ratio of 2:1, and the mixture was washed in saline (0.9% NaCl). An aliquot of 30 μl of the mixture was spotted on an LB plate incubated at 37°C for 24 h and plated on M9 agar plates (6.78 g/L Na_2_HPO_4_, 3 g/L KH_2_PO_4_, 1 g/L NH_4_Cl, 0.5 g/L NaCl, 20% glucose, 1.5% agar) with ampicillin (100 μg/ml) and kanamycin (25 μg/ml) for selection of transconjugants. Several of the ampicillin and kanamycin-resistant transconjugants were picked, grown in LB broth supplemented with 500 μg/ml streptomycin to exponential phase at 37°C, and then spread on LB agar plate containing 500 μg/ml streptomycin. Following the occurrence of a double crossover, streptomycin-resistant and kanamycin-sensitive colonies were selected and the deletion was verified by PCR and Southern hybridization (data not shown).

To obtain the complementation plasmid (pOmpR), a DNA fragment containing the promoter and coding sequence of *ompR* was PCR amplified using primer pair SY01/SY02 (**Table 2**) and cloned into the pACYC184 shuttle vector (Chang and Cohen, 1978). Next, pompR and pACYC184 were transformed into Δ*ompR* strain by electroporation.

### Construction of a *K. pneumoniae ompR_D55A_* Mutant

A DNA fragment carrying *ompR* and approximately 1000 bp adjacent regions on either side was amplified by PCR using primer pairs GT257/GT357 (**Table 2**) and cloned into yT&A vector (Yeastern). The resulting plasmid was used as the template for the inverse-PCR (Ochman et al., 1988) with the primer pair GT356/GT363 (**Table 2**) to generate a mutant *ompR* allele encoding the D55A mutation, which was confirmed by Sanger sequencing (Genomics, Taiwan). Subsequently, the mutant allele of *ompR* was subcloned into pKAS46 (Skorupski and Taylor, 1996) and confirmed by DNA sequencing. Then, the plasmid was mobilized from *E. coli* S17-1 λ*pir* to the *K. pneumoniae* Δ*ompR* strain by conjugation, and the subsequent selection was performed as described above.

### Western Blotting

The total proteins of exponential phase *K. pneumoniae* cultures were separated by sodium dodecyl sulfate polyacrylamide gel electrophoresis (SDS-PAGE) (approximately 5 μg per lane) and transferred to polyvinylidene difluoride membrane. Western analysis was followed as previously described (Lin et al., 2016). Rabbit anti-MrkA antibody (customer antibody service from LTK BioLaboratories) and Goat anti-rabbit immunoglobulin G antibody conjugated to horseradish peroxidase (Abcam) were used as the primary antibody and the secondary antibody, respectively. After incubation with the secondary antibody, the signal in the membranes was collected by ImageQuant LAS 4000 mini (GE Health, United States) after the visualization with an enhanced chemiluminescence ECL western blotting luminal reagent (PerkinElmer, Wellesley, MA, United States).

### Quantitative Reverse-Transcription PCR (qRT-PCR)

As previous study, total RNA extraction, reverse transcription of isolated mRNA to cDNA, qRT-PCR, and data analysis were performed (Lin et al., 2013). Primers and probes for selected target sequences were designed by using Universal ProbeLibrary Assay Design Center (Roche-applied science) and shown in **Table 2**. Relative gene expressions were quantified using the comparative threshold cycle 2^-ΔΔCT^ method with 23S rRNA as the endogenous reference.

### Measurement of Promoter Activity

To measure the *mrkA* promoter activity, *K. pneumoniae* strains was transformed with the promoter-reporter plasmid pmrkAZ15 into by electroporation, respectively. As previously described (Lin et al., 2006), the β-galactosidase activity of logarithmic phase bacteria was measured.

### Construction and Purification of OmpR::His_6_

The coding region of *ompR* was PCR amplified with primer sets GT336/GT337 (**Table 2**) and cloned into the *Nde*I/*Hind*III site in pET30b (Novagen, 205 Madison, Wis). The resulting plasmid pET30b-OmpR was then transformed into *E. coli* BL21(DE3) (New England Biolabs), and overproduction of the recombinant protein was induced by the addition of 0.1 mM isopropyl β-D-1-thiogalactopyranoside (IPTG) for 4 h at 37°C. The recombinant proteins were then purified from the soluble fraction of the total cell lysate by affinity chromatography using His-Bind resin (Novagen, Madison, Wis). Finally, the purified proteins were dialyzed against GMS buffer (50 mM Tris-HCl, pH 7.5, 50 mM KCl, 10 mM MgCl_2_, 0.5 mM EDTA, and 10% glycerol) at 4°C overnight, and the purity was determined by SDS-PAGE.

### Electrophoretic Mobility Shift Assay (EMSA)

DNA fragments of the putative promoter region of *mrkA*, *mrkHI*, *mrkJ*, and *ompF* were individually PCR-amplified with primer pairs CC323/CC324, GT288/GT290, GT284/GT285, and GT367/GT368 sets by Pfu-polymerase to generate DNA probes for EMSA. Briefly, the purified OmpR::His_6_ was incubated with 10 ng DNA in a 10 μl solution containing 10 mM Tris-HCl, pH 7.4, 50 mM KCl, 1 mM DTT, 10 mM MgCl_2_, 10% glycerol, and 25 mM acetyl-phosphate at room temperature for 20 min. The samples were then loaded onto a native gel of 5% non-denaturing polyacrylamide in 0.5X TB buffer (45 mM Tris-HCl, pH 8.0, 45 mM boric acid). Gels were electrophoresed with a 20-mA current at 4°C and then stained with SYBR Green I dye (Invitrogen). The assay was repeated in at least 3 independent experiments.

### Intracellular Concentration of c-di-GMP

The intracellular c-di-GMP in late exponential phase *K. pneumoniae* cultures (OD_600nm_ = 1.0–1.2) was extracted according to the previous study (Morgan et al., 2006). The dried extracts were solubilized in distill water and further were measured the c-di-GMP level by a ELISA kit (Wuhan EIAab Science Co., Ltd). The c-di-GMP concentration was normalized by total protein concentration. Relative percentage of c-di-GMP content was calculated by the c-di-GMP concentration of extracts is relative to that of wild type (WT) strain.

### PDE Activity

The PDE activity of the crude extracts in exponential phase *K. pneumoniae* strains was performed as previous study by using *bis*(*p*-nitrophenyl) phosphate (*bis*-*p*NPP) (Johnson and Clegg, 2010). Briefly, 10 μg of total protein in assay buffer (50 mM Tris-HCl, 1 mM MnCl_2_ [pH 8.5]) supplemented with 5 mM bis-*p*NPP at 37°C for 5 min. The PDE activity was determined by measuring the release of *p*-nitrophenol at OD_410nm_. Relative percentage of PDE activity was calculated by the OD_410nm_ of crude extracts is relative to that of WT strain.

### Quantification of Biofilm Amount

Biofilm amount was assessed by the ability of the cells to adhere to the walls of 96-well microtitre dishes made of PVC (TPP 96 flat) with some modification of the reported protocol (Lembke et al., 2006). The plate contained an aliquot of 1:10 diluted overnight bacteria culture and then was incubated at 37°C statically for 24 h for biofilm formation. The biofilms were washed triply with 200 μl PBS to remove non-adherent bacteria and then adherent bacteria was stained with 200 μl of 0.1% safranin solution at room temperature for 30 min. The plates was rinsed twice with deionizer water to remove excess stain. Finally, the safranin stained biofilm was solubilized in 200 μl of 95% ethanol and the absorbance determined at OD_492nm_.

### Statistical Method

The results of qRT-PCR analysis, promoter activity, c-di-GMP concentration, PDE activity, and biofilm amount were performed by biological replicates at least triplicate. The results are showed as the mean and standard deviation. Differences between groups were evaluated by an unpaired *t*-test. Values of *P* < 0.05 and *P* < 0.01 were considered statistically significant differences.

## Results

### The Role of OmpR in Regulating MrkA Production Under Hypertonic Conditions

**FIGURE 1:**
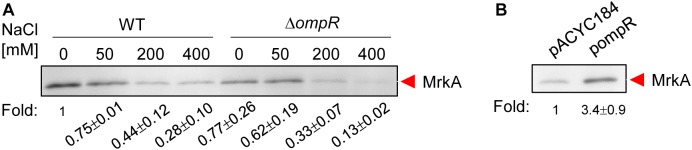
Hypertonic stress and OmpR affect MrkA production ount in *K. pneumoniae* CG43S3. **(A)** *K. pneumoniae* CG43S3 WT and Δ*ompR* strains were grown overnight at 37°C with agitation in LB broth with or without NaCl as indicated to observe the MrkA production by western blot analysis against MrkA. Likewise, the strains, Δ*ompR* (pACYC184) and Δ*ompR* (pOmpR)_,_ were grown to mid-log phase at 37°C in LB broth with 400 mM NaCl to observe the MrkA production **(B)**. The fold change of MrkA production calculated by Image J software is also shown.

To investigate whether the expression of type 3 fimbriae in *K. pneumoniae* is affected by hypertonic conditions, *K. pneumoniae* CG43S3 was grown in LB broth with increasing concentrations of NaCl and the MrkA production, the major subunit of type 3 fimbriae, was determined by western blotting analysis. As shown in **Figure 1A**, MrkA production decreased when *K. pneumoniae* was grown in LB broth with 50, 200, and 400 mM NaCl, compared to LB broth without NaCl. This indicates that MrkA production was repressed in response to increasing hypertonic stress. To determine whether OmpR is involved in this regulation, a deletion mutant of *ompR* was generated in *K. pneumoniae* CG43S3. MrkA production similarity decreased in the Δ*ompR* strain in response to NaCl. However, MrkA production was lower in the Δ*ompR* strain than in the WT strain in LB broth without NaCl; in LB broth with 400 mM NaCl, MrkA production was markedly reduced in the Δ*ompR* strain compared to the WT strain. To confirm the effect of *ompR* deletion on MrkA production, the complementation plasmid, pompR, and the empty vector, pACYC184, were introduced into the Δ*ompR* strain and MrkA production was evaluated in LB broth with 400 mM NaCl. As shown in **Figure 1B**, MrkA production was higher in the Δ*ompR*[pompR] strain than in the Δ*ompR*[pACYC184] strain; this confirms that OmpR can increase MrkA production in *K. pneumoniae* CG43S3 in response to hypertonic stresses.

### OmpR Increases *mrkA* Expression at the Transcriptional Level

**FIGURE 2:**
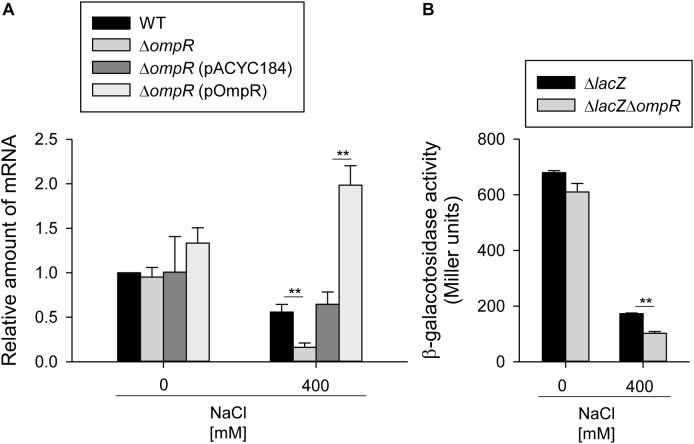
OmpR activates *mrkA* expression. **(A)** qRT-PCR analyses of the *mrkA* expression for WT, Δ*ompR*, Δ*ompR* (pACYC184), and Δ*ompR* (pOmpR) strains in LB broth with or without 400 mM NaCl. **(B)** β-galactosidase activities of *K. pneumoniae* CG43S3Δ*lacZ* and the isogenic strain (Δ*lacZ*Δ*ompR*) carrying the reporter plasmid pmrkAZ15 (P*_mrkA_*::*lacZ)* were determined using log-phase cultures grown in LB broth with or without 400 mM NaCl. The results are representative of three independent experiments. Error bars indicate standard deviations. ^∗∗^*P* < 0.01 compared to the indicated group.

To analyze the regulatory role of OmpR in type 3 fimbriae expression in response to hypertonic stimulus, the effect of *ompR* deletion on *mrkA* mRNA levels was measured in the WT and Δ*ompR* strains grown in LB broth with or without 400 mM NaCl using qRT-PCR. As shown in **Figure 2A**, *mrkA* mRNA levels were lower in the ΔtextitompR strain than in the WT strain in LB broth with 400 mM NaCl, while no apparent effect was observed in LB without NaCl. In addition, introduction of the complementation plasmid pompR into Δ*ompR* reversed the effect of the deletion in response to 400 mM NaCl. To further investigate whether OmpR affects the promoter activity of *mrkA*, a plasmid carrying P*_mrkA_* fused to a *lacZ* reporter gene was introduced into the Δ*lacZ* and Δ*lacZ*Δ*ompR* strains. As shown in **Figure 2B**, P*_mrkA_* activity was significantly lower in Δ*lacZ*Δ*ompR* than in Δ*lacZ*, suggesting that OmpR increases type 3 fimbriae expression at the transcriptional level in response to hypertonic stresses.

### Phosphorylated OmpR Is Required for Increasing MrkA Expression

**FIGURE 3:**
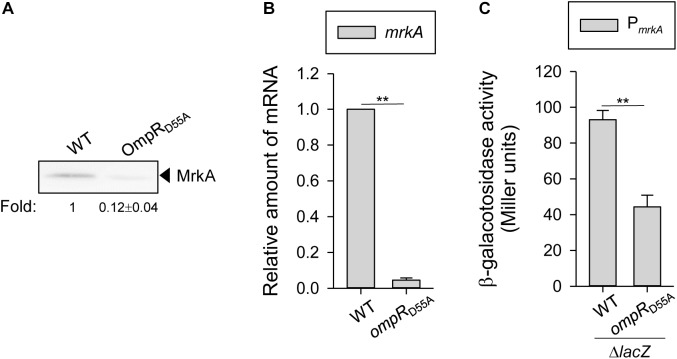
Effect of phosphorylated OmpR on regulating MrkA expression in *K. pneumoniae*. *K. pneumoniae* CG43S3 WT and *ompR*_D55A_ strains was grown to mid-log phase at 37°C in LB broth with 400 mM NaCl to observe the type 3 fimbriae expression by **(A)** western blot analysis against MrkA, and **(B)** qRT-PCR analyses of *mrkA* gene expression. **(C)** β-galactosidase activities of *K. pneumoniae* CG43S3 Δ*lacZ* and the isogenic strains (Δ*lacZ*_OmpR_D55A_) carrying the reporter plasmid pmrkAZ15 (P*_mrkA_*::*lacZ*) were determined using log-phase cultures grown in LB broth with 400 mM NaCl. The results are representative of three independent experiments. Error bars indicate standard deviations. ^∗∗^*P* < 0.01 compared to the indicated group. The fold change of MrkA production calculated by Image J software is also shown.

To test whether the effect of OmpR on *mrkA* transcription and protein production might require phosphorylation of OmpR at aspartate 55, a site-directed mutant with a single amino acid substitution of aspartate 55 to alanine (D55A), which prevents phosphorylation, was generated. As shown in **Figure 3A**, MrkA production was decreased in the *ompR*_D55A_ mutant than in the WT strain when grown in LB broth with 400 mM NaCl. Furthermore, *mrkA* mRNA levels were lower in the *ompR*_D55A_ mutant compared to the WT strain when grown in LB broth with 400 mM NaCl (**Figure 3B**). Similarly, lower *mrkA* promoter activity was observed in the OmpR_D55A_ mutant (**Figure 3C**). These results suggest that the phosphorylated form of OmpR is required for increasing type 3 fimbriae expression.

### OmpR-Mediated Regulation of Type 3 Fimbriae Expression Is Probably Indirect

**FIGURE 4:**
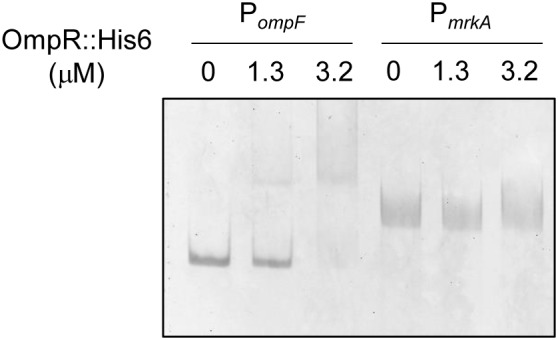
Binding activity of phosphorylated OmpR::His_6_ with P*_ompF_* and P*_mrkA_*. Different concentrations of purified OmpR::His_6_ were incubated with 25 mM acetyl phosphate and 10 ng of the upstream regions of *ompF* and *mrkA*. Following incubation at room temperature for 20 min, the mixtures were analyzed on a 5% non-denaturing polyacrylamide gel. The gel was stained with SYBR Green I dye and photographed.

To investigate the mechanism underlying OmpR regulation of *mrkA* transcription, the consensus sequence of the *E. coli* OmpR∼P binding site was used as a reference for analyzing the *mrkA* promoter sequence (Yoshida et al., 2006). However, no typical OmpR binding site was found upstream of *mrkA*. To further confirm the absence of OmpR∼P binding activity in P*_mrkA_*, the interaction between a recombinant OmpR::His_6_ protein phosphorylated by acetyl-phosphate and DNA fragments containing P*_mrkA_* and P*_ompF_* (positive control) were examined by EMSA. As shown in **Figure 4**, a DNA-protein-binding complex was observed following incubation of 1.3 μM phosphorylated OmpR::His_6_ and 10 ng P*_ompF_*. However, no binding activity was observed for the phosphorylated OmpR::His6 protein with P*_mrkA_* (**Figure 4**). These results indicate that OmpR activation of type 3 fimbriae expression may be indirect.

### Regulatory Effect of OmpR on *mrkHIJ* Expression

**FIGURE 5:**
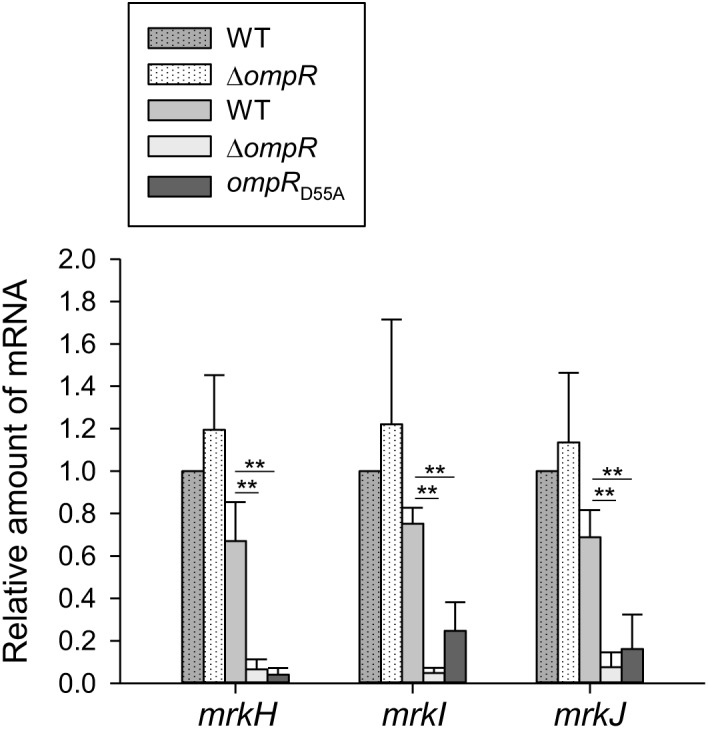
Effect of OmpR on regulating *mrkH*, *mrkI*, and *mrkJ* expression. qRT-PCR analyses of the *mrkH*, *mrkI*, and *mrkJ* expressions for WT, Δ*ompR*, and *ompR*_D55A_ strains in LB broth with (solid bars) or without 400 mM NaCl (dot bars). The results are representative of three independent experiments. Error bars indicate standard deviations. ^∗^*P* < 0.05 and ^∗∗^*P* < 0.01 compared to the indicated group.

Previous studies have demonstrated that MrkHIJ play an important role in regulating type 3 fimbriae expression (Murphy and Clegg, 2012; Wu et al., 2012). To further examine whether MrkHIJ are involved in OmpR regulon, the mRNA levels of these genes were measured in the WT and Δ*ompR* strains grown in LB broth with or without 400 mM NaCl by qRT-PCR. In contrast to the lack of an apparent effect in the WT and Δ*ompR* strains in LB without NaCl, *mrkH*, *mrkI*, and *mrkJ* mRNA levels were markedly decreased in the Δ*ompR* strain in LB broth with 400 mM NaCl (**Figure 5**). In addition, a similar reduction was also observed in the *ompR*_D55A_ mutant. This indicates that the phosphorylated form of OmpR is required for increasing *mrkH*, *mrkI*, and *mrkJ* mRNA expression. However, no typical OmpR∼P binding site was found upstream of *mrkHI* and *mrkJ* and no binding activity was observed for the phosphorylated OmpR::His6 protein with P*_mrkHI_* and P*_mrkJ_* (data not shown), suggesting that OmpR∼P positive regulation of *mrkH*, *mrkI*, and *mrkJ* expression is indirect.

### Effect of OmpR on Intracellular c-di-GMP Concentration, PDE Activity, and PDE-Related Genes Expression

**FIGURE 6:**
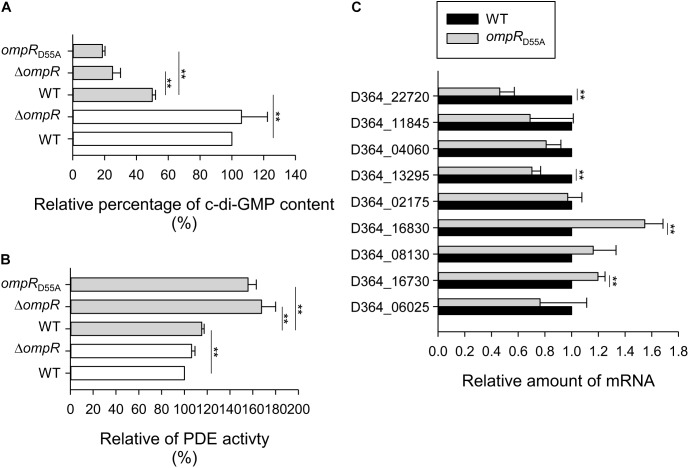
Effect of phosphorylated OmpR on c-di-GMP concentration, PDE activity, and PDE-related genes expression. **(A)** c-di-GMP concentration of WT, Δ*ompR*, and *ompR*_D55A_ strains in LB broth with 400 mM NaCl (solid gray bars) or without (open white bars) was quantified by ELISA according to the manual (Wuhan EIAab Science). Relative percentage of c-di-GMP content was calculated by the c-di-GMP concentration of extracts is relative to that of WT strain. **(B)** The PDE activity of crude extracts of WT, Δ*ompR*, and *ompR*_D55A_ strains in LB broth with 400 mM NaCl (solid gray bars) or without (open white bars) was determined by using *bis*-pNPP as substrate and measuring the absorbance at OD410. Relative percentage of PDE activity was calculated by the OD410 of crude extracts is relative to that of WT strain. **(C)** qRT-PCR analyses of the PDE-related genes expression for WT and *ompR*_D55A_ strains in LB broth with 400 mM NaCl. The results are representative of three independent experiments. Error bars indicate standard deviations. ^∗∗^*P* < 0.01 compared to the indicated group.

Based on the previous study, the expression of type 3 fimbriae and MrkHI is c-di-GMP dependent (Tan et al., 2015). To further investigate whether OmpR affects the c-di-GMP production, thus increasing type 3 fimbriae and MrkHI expression, the intracellular concentration of c-di-GMP was measured in the WT and Δ*ompR* strains grown in LB broth with or without 400 mM NaCl. As shown in **Figure 6A**, the c-di-GMP concentration of *K. pneumoniae* decreased in LB with 400 mM NaCl. Furthermore, the c-di-GMP concentration was lower in the Δ*ompR* strain than in the WT strain in LB broth with 400 mM NaCl, while this effect was not observed in LB without NaCl. An apparent lower intracellular c-di-GMP concentration was also found in the *ompR*_D55A_ strain relative to the WT strain when grown in LB with 400 mM NaCl. These results indicate that OmpR∼P is required for increasing intracellular c-di-GMP production in *K. pneumoniae* under hypertonic conditions. In bacteria, intracellular c-di-GMP is degraded by PDEs (Simm et al., 2004; Hengge, 2009). Therefore, we evaluated the *in vitro* PDE activity of crude extracts from the WT and Δ*ompR* strains grown in LB with or without 400 mM NaCl using a colorimetric assay. As shown in **Figure 6B**, the WT strain demonstrated a slightly higher PDE activity in LB with 400 mM NaCl. Furthermore, the PDE activity in the Δ*ompR* extract was higher than in the WT extract in LB with 400 mM NaCl, but not in LB without NaCl. In addition, the *ompR*_D55A_ mutant exhibited higher PDE activity in response to 400 mM NaCl compared to the WT strain. Therefore, we hypothesized that OmpR∼P can decrease PDE activity to further increase intracellular c-di-GMP concentrations, resulting in elevated type 3 fimbriae and *mrkHI* expression. Analysis of the upstream sequences of the c-di-GMP PDE related open reading frames (ORFs) in *K. pneumoniae* CG43, including *mrkJ* (D364_16630), D364_06025, *fimK* (D364_16730), D364_04060, D364_08130, D364_16830, *ylaB* (D364_02175), *yoaD* (D364_11845), *rtn* (D364_13295), and *yjcC* (D364_22720), did not reveal any typical OmpR∼P binding sites (data not shown). To further investigate the effect of OmpR∼P on the expression of these c-di-GMP PDE related ORFs, the mRNA expression of these ORFs was measured in the WT and *ompR*_D55A_ strains in response to 400 mM NaCl. As shown in **Figure 6C**, the mRNA levels of *fimK* and D364_16830 were increased, while the mRNA levels of D364_13295 and *yjcC* were decreased in the *ompR*_D55A_ strain. Therefore, *fimK* and D364_16830 may be involved in repression of PDE activity by OmpR∼P.

### OmpR Increases Biofilm Amount Under Hypertonic Conditions

**FIGURE 7:**
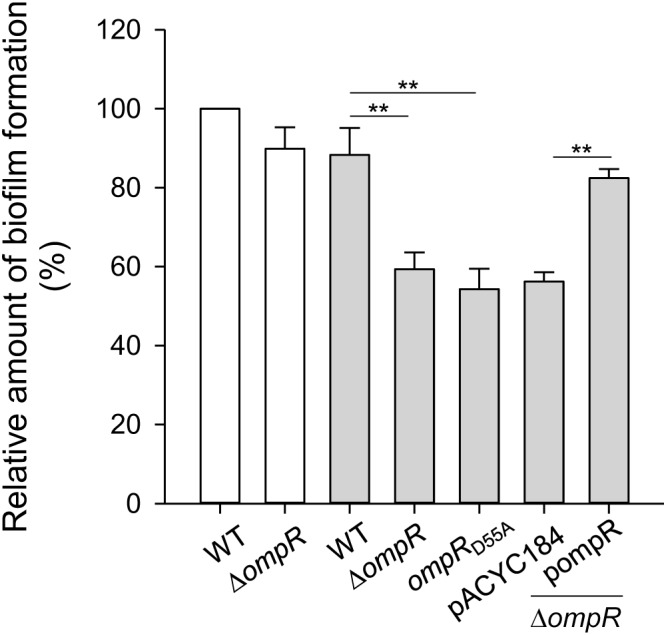
Effect of OmpR on biofilm formation. *K. pneumoniae* strains CG43S3 WT, Δ*ompR*, *ompR*_D55A_, Δ*ompR* (pACYC184), and Δ*ompR* (pOmpR) were grown at 37°C for 24 h in LB broth with 400 mM NaCl (solid gray bars) or without (open white bars), and bacterial biofilm formation was quantified as described in Materials and Methods. The results are representative of three independent experiments. Error bars indicate standard deviations. ^∗∗^*P* < 0.01 compared to the indicated group.

Type 3 fimbriae are a major determinant modulating *K. pneumoniae* biofilm formation (Di Martino et al., 2003; Jagnow and Clegg, 2003; Wu et al., 2012). To further investigate the role of OmpR in *K. pneumoniae* biofilm formation, the biofilm amount of WT and Δ*ompR* grown in LB broth with and without 400 mM NaCl was measured. As shown in **Figure 7**, biofilm amount was lower in Δ*ompR* than in the WT strain in LB broth with 400 mM NaCl, while no apparent effect was observed in LB broth without NaCl. Furthermore, introduction of the complementation plasmid, pompR, into the Δ*ompR* strain increased biofilm amount, compared to Δ*ompR* carrying the empty vector in LB broth supplemented with 400 mM NaCl. These results indicate that OmpR can increase biofilm amount in *K. pneumoniae* under hypertonic conditions. In addition, we evaluated biofilm amount in the *ompR*_D55A_ mutant grown LB broth supplemented with 400 mM NaCl; biofilm amount was decreased in the *ompR*_D55A_ strain compared to the WT strain, indicating that the phosphorylated form of OmpR increased biofilm amount in *K. pneumoniae* CG43S3 under hypertonic conditions.

## Discussion

OmpR/EnvZ is a well-known TCS in many species of *Enterobacteriaceae*, which regulates the expression of various virulence genes in response to osmotic stresses (Mizuno and Mizushima, 1990; Oshima et al., 2002). However, the role of OmpR/EnvZ in *K. pneumoniae* pathogenesis remains largely unknown. In this study, we found that OmpR∼P activated type 3 fimbriae expression and biofilm formation under hypertonic conditions. Additionally, OmpR regulation of type 3 fimbriae expression appeared to involve the c-di-GMP signaling pathway. Our previous studies have also shown that transcription factors, such as Fur, cAMP-CRP, and IscR, coordinate c-di-GMP related proteins, MrkH, MrkI, and MrkJ, to regulate type 3 fimbriae expression in response to iron availability and exogenous glucose stimuli in *K. pneumoniae* (Wu et al., 2012; Lin et al., 2016, 2017). These findings indicate that *K. pneumoniae* needs to orchestrate various signaling pathways in response to dynamic environmental cues in order to regulate the expression of virulence factors for successful infection.

In this study, we found that osmotic stress repressed type 3 fimbriae expression in *K. pneumoniae* CG43S3 (**Figure 1A**). In *E. coli*, transcriptional regulators, including CpxAR, RpoS, Lrp, and H-NS, have been demonstrated to play important roles in bacterial responses to osmotic stress (Levinthal and Pownder, 1996; De Wulf et al., 1999; Giuliodori et al., 2007; Hengge, 2008; Pruss, 2017). Of these regulators, H-NS has been shown to directly repress the expression of *mrkHIJ*, while activating the expression of *mrkA* (Ares et al., 2016, 2017). It has also been demonstrated that increased osmolality can reduce the polymerization of H-NS tetramers and subsequently decrease the regulatory activity of H-NS (Stella et al., 2006). Thus, whether polymerization of H-NS in response to osmotic stress affects type 3 fimbriae expression in *K. pneumoniae* should be investigated in future studies. Furthermore, we noted that OmpR activated type 3 fimbriae expression under hypertonic conditions and that the phosphorylation status of OmpR played a crucial role in this regulation (**Figures 1**, **3**). Intracellular OmpR∼P levels are tightly controlled by EnvZ, which exhibits high phosphatase activity and low kinase activity at low osmolality (Mattison and Kenney, 2002). Taken together, these findings suggest that the relatively high level of OmpR∼P present in response to hypertonic conditions can increase type 3 fimbriae expression in *K. pneumoniae*.

In *K. pneumoniae*, the type 1 and type 3 fimbrial gene clusters are physically linked and deletion of *mrkA* increases the production of FimA, the major subunit of type 1 fimbriae (Wang et al., 2013), implying that expression of these fimbriae is coordinately regulated. In *E. coli*, deletion of *ompR* increased type 1 fimbriae expression via the activation of *fimB* transcription (Rentschler et al., 2013). We also found that the mRNA levels of *fimA* and *fimB* were increased in the *K. pneumoniae* Δ*ompR* strain compared to the WT strain when grown in LB broth with 400 mM NaCl (**Supplementary Figure S1**). This result shows that OmpR also decreases type 1 fimbriae expression in *K. pneumoniae*; further investigation is required to elucidate the underlying mechanism(s). To our knowledge, the gene clusters, *mrkABCDEF* and *mrkHIJ*, are highly conserved among *K. pneumniae* strains with about 99% amino acid identities, but are not found in other bacteria. Since we focused on the study of the OmpR regulation on the expression of *mrk* genes, which are not found in the genomes of *S.* Typhimurium, *S. flexneri*, *Y. pestis*, and *A. baumanni*, it is difficult to make a comparison.

High concentrations of c-di-GMP are linked to bacterial biofilm formation (Caly et al., 2015; Jenal et al., 2017). In *K. pneumoniae*, intracellular c-di-GMP concentrations influence the regulatory activity of MrkH on type 3 fimbriae expression and bacterial biofilm amount (Wilksch et al., 2011; Yang et al., 2013). Our results might indicate that OmpR indirectly regulates the expression of *mrkHIJ* (**Figure 5**). Multiple genes encoding GGDEF- and EAL-domain containing proteins have been identified in the *K. pneumoniae* genome and their expression, in response to various environmental stimuli, may influence intracellular c-di-GMP concentrations (Ferreira et al., 2008; Kalia et al., 2012). Although OmpR∼P has an apparent effect on modulating c-di-GMP concentration and the intracellular PDE activity, only a slight increase in the mRNA levels of *fimK* and D364_16830 was observed (**Figure 6**). Whether OmpR∼P affects the expression of *fimK* and D364_16830 to increase PDE activity, needs to be clarified. In addition, hybrid proteins with both GGDEF and EAL domains may exert dual activities or only single activity depending on various cellular conditions (Ferreira et al., 2008; Kalia et al., 2012). Therefore, OmpR may also affect the expression of ORFs encoding hybrid proteins with both GGDEF and EAL domains, to modulate PDE activity; this possibility needs to be further investigated. In *E. coli*, OmpR∼P represses the expression of *bolA*, which is responsible for cell growth and division (Yamamoto et al., 2000). Deletion of *bolA* increases intracellular c-di-GMP concentrations and biofilm formation in *E. coli* (Moreira et al., 2017), suggesting that OmpR∼P represses *bolA* expression to modulate intracellular c-di-GMP concentrations. We identified a *bolA* homolog (D364_02025) in the genome of *K. pneumoniae* CG43, which shared 91.3% amino acid sequence identity with the *E. coli* protein. However, determining whether OmpR regulates the expression of *bolA* to affect intracellular c-di-GMP concentrations, type 3 fimbriae production, and biofilm formation in *K. pneumoniae* will require further investigation.

Type 3 fimbriae expression is thought to play a crucial role in *K. pneumoniae* biofilm formation; thus, we hypothesized that bacterial biofilm formation is affected by osmotic stimuli. However, no apparent effect on biofilm formation was observed when *K. pneumoniae* was grown in LB broth with or without 400 mM NaCl (**Figure 7**). In addition to type 3 fimbriae, multiple factors have been reported to affect *K. pneumoniae* biofilm formation such as CPS, LPS, quorum-sensing systems, and antibiotic resistance (Balestrino et al., 2005, 2008; Boddicker et al., 2006; Alcantar-Curiel et al., 2013; Khodadadian et al., 2017; Vuotto et al., 2017). We also found that CPS levels were increased under hypertonic conditions (**Supplementary Figure S2**). As the thick capsule of *K. pneumoniae* impedes the assembly of type 3 fimbriae (Schembri et al., 2005), the decrease in type 3 fimbriae under hypertonic conditions may also be due to an increase in CPS levels. However, no apparent difference in CPS amount was observed between WT and Δ*ompR* strains grown under hypertonic conditions (data not shown), implying the involvement of other regulator(s). In *E. coli*, OmpR regulates the expression of the porins OmpF and OmpC in response to osmotic stress (Mizuno and Mizushima, 1990). Furthermore, the deletion of *ompF* can increase bacterial biofilm formation (Yang et al., 2008). *K. pneumoniae* possess two classical porins, OmpK35 and OmpK36, which are homologs of *E. coli* OmpF and OmpC, respectively (Sugawara et al., 2016). In *K. pneumoniae*, biofilm formation is highly related to antibiotic resistance (Vuotto et al., 2017), which is affected by the expression of porins and efflux pumps (e.g., AcrAB) (Bialek et al., 2010; Pitout et al., 2015). Taken together, these findings suggest that OmpR∼P coordinately regulates the expression of OmpK35, OmpK36, and type 3 fimbriae to mediate *K. pneumoniae* biofilm formation. This possibility needs to be further investigated.

In this study, we provide evidence that OmpR∼P participates in the regulation of type 3 fimbriae and *mrkHIJ* expression, c-di-GMP concentration, and biofilm formation in response to osmotic stress, which may play an important role during successful infection.

## Author Contributions

T-HL, J-TK, YC, and C-TL conceived and designed the experiments. YC, J-TK, and C-TL performed the experiments. YC, J-TK, Y-CL, and C-TL analyzed the data. T-HL, Y-CL, C-CW, C-FH, and C-TL contributed reagents, materials, and analysis tools. T-HL and C-TL wrote the paper. All authors read and approved the final manuscript.

## Conflict of Interest Statement

The authors declare that the research was conducted in the absence of any commercial or financial relationships that could be construed as a potential conflict of interest.
