# Supplementary material for: Phosphorylated OmpR Is Required for Type 3 Fimbriae Expression in Klebsiella pneumoniae Under Hypertonic Conditions
Source: Front Microbiol. 2018 Oct 12;9:2405. doi: 10.3389/fmicb.2018.02405 (PMC6194325; doi:10.3389/fmicb.2018.02405)
Supplement: Supplementary file 1 [file Presentation_1.PDF]

Supplementary Materials:

OmpR Regulates Type 3 Fimbriae Expression in *Klebsiella pneumoniae* Under Hypertonic Conditions

Tien-Huang Lin, Cheng-Yin Tseng, Yi-Chyi Lai, Chien-Chen Wu, Chun-Fa Huang, Ching-Ting Lin*

*** Correspondence:**
Ching-Ting Lin
gingting@mail.cmu.edu.tw

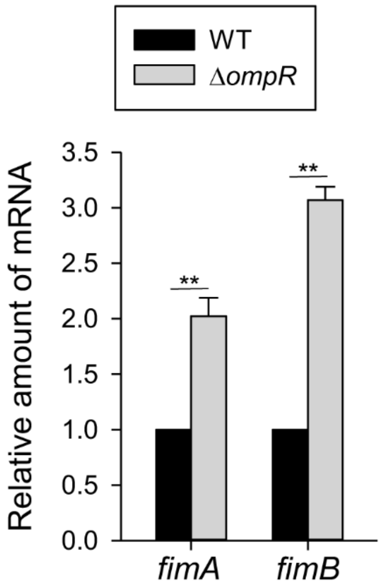

Figure S1. qRT-PCR analyses of *fimA* and *fimB* expression in *K. pneumoniae* CG43S3 WT and Δ*ompR strains* which were grown in LB medium with 400 mM NaCl.

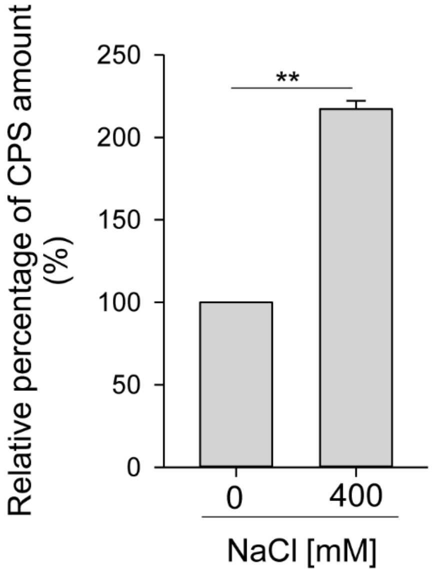

Figure S2. CPS amount of *K. pneumoniae* CG43S3 in response to osmotic stress.
